# Inherited disorders of cobalamin metabolism in childhood: biochemical and clinical perspectives

**DOI:** 10.3389/fnut.2026.1808765

**Published:** 2026-05-04

**Authors:** Arushi Gahlot Saini, Pradeep Kumar Gunasekaran, Asuri Narayan Prasad

**Affiliations:** 1 Department of Pediatrics, Postgraduate Institute of Medical Education and Research, Chandigarh, India; 2 Department of Pediatrics, NYC Health + Hospitals/Harlem, New York, NY, United States; 3 Department of Pediatrics and Clinical Neurological Sciences, Schulich School of Medicine and Dentistry, London, ON, Canada

**Keywords:** children, cobalamin, homocystinuria, inborn errors of metabolism, methylmalonic aciduria, vitamin B12

## Abstract

Cobalamin (vitamin B_12_) is a vitamin with a defined role in human metabolism. Since its discovery in the 20^th^ century, our understanding of its deficiency that results in multifaceted disorders with a significant impact on neurological health has evolved. While classically associated with megaloblastic anemia, its neurological manifestations can be diverse, severe, and often precede hematological changes. This review provides a detailed analysis of the role of cobalamin in different biochemical pathways, clinical syndromes, underlying pathophysiological mechanisms, and inborn errors of metabolism associated with genetic defects in the pediatric population. The primary neurological insult is related to demyelination and axonal loss in both the central and peripheral nervous systems, leading to a spectrum of symptoms from peripheral neuropathy to severe myelopathy and neuropsychiatric decline. The metabolic networks involve critical biochemical pathways affecting the methionine-homocysteine cycle, folate biosynthesis, and energy and lipid metabolism. The genetic basis of these disorders is defined by distinct complementation groups and the genetic mutations identified by molecular genetic testing. The clinical aspects often present with unique profiles and require specialized diagnostic approaches. A nuanced diagnostic strategy that includes measurement of methylmalonic acid and homocysteine is essential, as is prompt and aggressive parenteral treatment to prevent irreversible neurological damage.

## 1 Introduction

Neurological disorders involving cobalamin (vitamin B₁₂) metabolism constitute an important group of treatable neurometabolic conditions. From fetal life through early childhood, cobalamin-dependent pathways are critical for myelination, neuronal maturation, and mitochondrial energy metabolism, rendering infants and young children particularly vulnerable to disturbances in cobalamin availability or intracellular processing. Advances in biochemical and molecular genetics have elucidated the complex mechanisms governing cobalamin absorption, cellular uptake, and intracellular conversion to its active cofactors methylcobalamin (MeCbl) and adenosylcobalamin (AdoCbl), which are essential for one-carbon metabolism and mitochondrial function.

Inborn errors of cobalamin metabolism (Table 1), classified into distinct complementation groups, typically present in the neonatal period or early infancy with developmental delay, encephalopathy, seizures, hypotonia, and progressive neurodevelopmental impairment, although later-onset and phenotypically variable presentations are increasingly recognized. Characteristic biochemical abnormalities include isolated or combined methylmalonic acidemia and/or hyperhomocysteinemia. While timely diagnosis and metabolic treatment can substantially improve outcomes, delayed recognition remains common, underscoring the need for heightened clinical awareness.

**Table 1:** Inherited disorders of cobalamin metabolism.

Complementation group/disease (MIM#)	Gene (MIM*)	Cellular step/pathway	Affected cofactor(s)	Biochemical signature	Inheritance
Early-onset phenotypes					
Intracellular metabolic defects - combined MMA and homocystinuria					
cblC (277400)	MMACHC (609831)	Early cytosolic processing	AdoCbl ↓ MeCbl ↓	↑MMA ↑Hcy, ↓Met	AR
cblD combined (277410)	MMADHC (611935)	Cytosol-mitochondria sorting	AdoCbl ↓ MeCbl ↓	↑MMA ↑Hcy	AR
cblF (277380)	LMBRD1 (612625)	Lysosomal export	AdoCbl ↓ MeCbl ↓	↑MMA ↑Hcy	AR
cblJ (614857)	ABCD4 (603214)	Lysosome → cytosol transport	AdoCbl ↓ MeCbl ↓	↑MMA ↑Hcy	AR
cblX (309541)	HCFC1 (300019)	Nuclear transcriptional regulation	AdoCbl ↓ MeCbl ↓	↑MMA ↑Hcy	XL
cblK	ZNF143 (603433)	Nuclear transcriptional regulation	AdoCbl ↓ MeCbl ↓	↑MMA ↑Hcy	AR
Intracellular metabolic defects – isolated MMA					
cblA (251100)	MMAA (607481)	Mitochondrial chaperone	AdoCbl ↓	↑MMA	AR
cblB (251110)	MMAB (607568)	Adenosyltransferase (mitochondrial)	AdoCbl ↓	↑MMA	AR
Intracellular metabolic defects – isolated homocystinuria (remethylation defects)					
cblE (236270)	MTRR (602568)	MeCbl regeneration (cytosol)	MeCbl ↓	↑Hcy ↓Met	AR
cblG (250940)	MTR (156570)	Methionine synthase (MeCbl-dependent)	MeCbl ↓	↑Hcy ↓Met	AR
Transport and absorption defects – early-onset					
Transcobalamin II deficiency (275350)	TCN2 (607666)	Plasma transport of cobalamin to cells	AdoCbl ↓ MeCbl ↓	↑MMA ↑Hcy	AR
CD320 deficiency (613646)	CD320 (613646)	Cellular uptake of holotranscobalamin via receptor-mediated endocytosis	AdoCbl ↓ MeCbl ↓	↑MMA ↑Hcy	AR
Imerslund–Gräsbeck syndrome (261100)	CUBN (602997)/AMN (605799)	Ileal absorption (IF-B12 complex)	AdoCbl ↓ MeCbl ↓	↑MMA and/or ↑Hcy	AR
Intrinsic factor deficiency	GIF (609342)	IF-B12 complex formation/ileal absorption	AdoCbl ↓ MeCbl ↓	↑MMA and/or ↑Hcy	AR
Complementation group/disease (MIM#)	Gene (MIM*)	Cellular step/pathway	Affected cofactor(s)	Biochemical signature	Inheritance
Late-onset phenotypes					
Intracellular metabolic defects – combined MMA and homocystinuria (late-onset)					
cblC late-onset (277400)	MMACHC (609831)	Early cytosolic processing	AdoCbl ↓ MeCbl ↓	↑MMA ↑Hcy, ↓Met	AR
cblD variant 1/variant 2 (277410)	MMADHC (611935)	Mutation-position dependent; N-terminal vs. C-terminal	AdoCbl ↓ or MeCbl ↓	↑MMA and/or ↑Hcy	AR
Intracellular metabolic defects – isolated homocystinuria (late-onset)					
cblE late-onset (236270)	MTRR (602568)	MeCbl regeneration (cytosol)	MeCbl ↓	↑Hcy ↓Met	AR
cblG late-onset (250940)	MTR (156570)	Methionine synthase (MeCbl-dependent)	MeCbl ↓	↑Hcy ↓Met	AR
Transport defects – late-onset					
Haptocorrin (TCN1) deficiency	TCN1 (607599)	Plasma B12 binding/transport (haptocorrin)	Usually normal intracellular	Altered serum B12 only; usually clinically silent	AR

AdoCbl, adenosylcobalamin; AR, autosomal recessive; Hcy, homocysteine; MeCbl, methylcobalamin; Met, methionine; MMA, methylmalonic acid; XL, X-linked.

This review integrates biochemical and clinical perspectives to delineate mechanisms of neurological injury in cobalamin-related disorders. This approach synthesizes the biochemistry of cobalamin-dependent pathways, the spectrum of neurological phenotypes across complementation groups, and a structured diagnostic approach incorporating biomarkers, neuroimaging, and genomic evaluation, alongside current therapeutic strategies and outcomes. The current paper focuses exclusively on the pediatric spectrum within the contemporary framework of complementation group classification and precision medicine and adds to the existing literature on inherited disorders of cobalamin metabolism.

## 2 Biochemistry of cobalamin

### 2.1 Chemical forms and sources

Cobalamin is derived almost exclusively from animal-based foods, including meat, fish, eggs, and dairy products, with fortified foods and supplements serving as important sources for individuals on plant-based diets. It is a water-soluble micronutrient with a unique cobalt-containing structure, essential for human metabolism. A central cobalt atom within a corrin ring enables reversible redox reactions critical for enzymatic function (1).

### 2.2 Intestinal absorption and transport

Dietary cobalamin is released from food proteins in the stomach by gastric acid and pepsin. It initially binds to haptocorrin (R-protein) secreted in saliva and gastric fluid (2). In the duodenum, pancreatic proteases degrade haptocorrin, thus allowing cobalamin to bind intrinsic factor (IF). This complex is then absorbed in the distal ileum via the calcium-dependent cubilin-amnionless (Cubam) receptor complex (3). Following absorption, cobalamin enters the circulation bound primarily to haptocorrin (≈80–90%), while transcobalamin (≈10–20%) carries the biologically active fraction responsible for tissue delivery. Only transcobalamin-bound cobalamin (holotranscobalamin) is available for cellular uptake, explaining why total serum B₁₂ may be misleading in functional deficiency.

### 2.3 Cellular uptake and intracellular trafficking

Cobalamin enters cells by receptor-mediated endocytosis of holotranscobalamin (transcobalamin-bound cobalamin) via the CD320 receptor and, in the kidney, via megalin (multiligand endocytic receptor) (4, 5). In lysosomes, carrier proteins are degraded, and cobalamin is moved out in the cytosol by the LMBD1-ABCD4 complex. LMBD1 (lysosomal cobalamin-binding protein) chaperones ABCD4 (an ATP-binding cassette subfamily D member 4 transporter) to the lysosomal membrane, and after assembly, the LMBD1-ABCD4 complex transports cobalamin from the lysosome into the cytosol.

The MMACHC enzyme in the cytosol removes upper ligands from incoming cobalamin and, together with MMADHC (adaptor protein), directs cobalamin toward synthesis of its two active coenzyme forms: AdoCbl and MeCbl. MeCbl functions in the cytosol as a cofactor for methionine synthase, supporting one-carbon metabolism, DNA synthesis, and cellular methylation (6). In mitochondria, MMAB (adenosyl transferase) catalyzes the synthesis of AdoCbl, while MMAA acts as a GTP-dependent chaperone that facilitates cofactor assembly and delivery to methylmalonyl-CoA mutase. AdoCbl then functions in the mitochondria as the essential cofactor for methylmalonyl-CoA mutase, required for odd-chain fatty acid and amino acid catabolism. Together, these pathways underpin myelin integrity, energy metabolism, and normal cellular proliferation, explaining the neurological vulnerability to cobalamin deficiency. The key foundational discoveries in inherited disorders of cobalamin metabolism are summarized in Supplementary Table 1.

### 2.4 Biochemical pathways of cobalamin metabolism

Cobalamin functions through cytosolic and mitochondrial enzymes to integrate one-carbon metabolism with energy homeostasis (7). Three key enzymes (8) involved in the process are listed below:

- Methionine synthase: Located in the cytosol, it uses MeCbl to remethylate homocysteine to methionine, regenerating tetrahydrofolate (THF) for nucleotide synthesis.
- Methionine synthase reductase: Located in the cytosol, it restores methionine synthase activity via reductive methylation of the cofactor. Methionine is a precursor for S-adenosylmethionine (SAM), the universal methyl donor.
- Methylmalonyl-CoA mutase: Located in mitochondria, it uses AdoCbl to convert L-methylmalonyl-CoA to succinyl-CoA, feeding the Krebs’ cycle and linking cobalamin to energy metabolism.

*One-carbon metabolism:* Cobalamin links the folate cycle, methionine cycle, and SAM cycle, integrating one-carbon transfer reactions essential for methylation and nucleotide biosynthesis (7, 9). In the cytosol, MeCbl-dependent methionine synthase transfers a methyl group from 5-methyltetrahydrofolate to homocysteine, regenerating THF for nucleotide synthesis. Methionine feeds the SAM cycle, providing universal methyl groups for DNA, RNA, histones, and protein methylation (10). Without functional methionine synthase due to cobalamin deficiency, SAM production plummets, leading to a state of global hypomethylation. This systemic loss of methylation, along with the buildup of toxic homocysteine, contributes significantly to the neurological and systemic pathologies observed in cobalamin deficiency (11).

*Folate trap in cobalamin deficiency:* The folate trap refers to the accumulation of 5-methyl THF when MeCbl-dependent methionine synthase is inactive due to cobalamin deficiency. THF is not regenerated, creating a functional folate deficiency that impairs nucleotide synthesis, particularly in rapidly dividing hematopoietic cells, resulting in megaloblastic anemia. Folic acid supplementation can temporarily restore DNA synthesis and correct anemia, but does not reverse neurological damage caused by persistent cobalamin deficiency.

**Figure 1:**
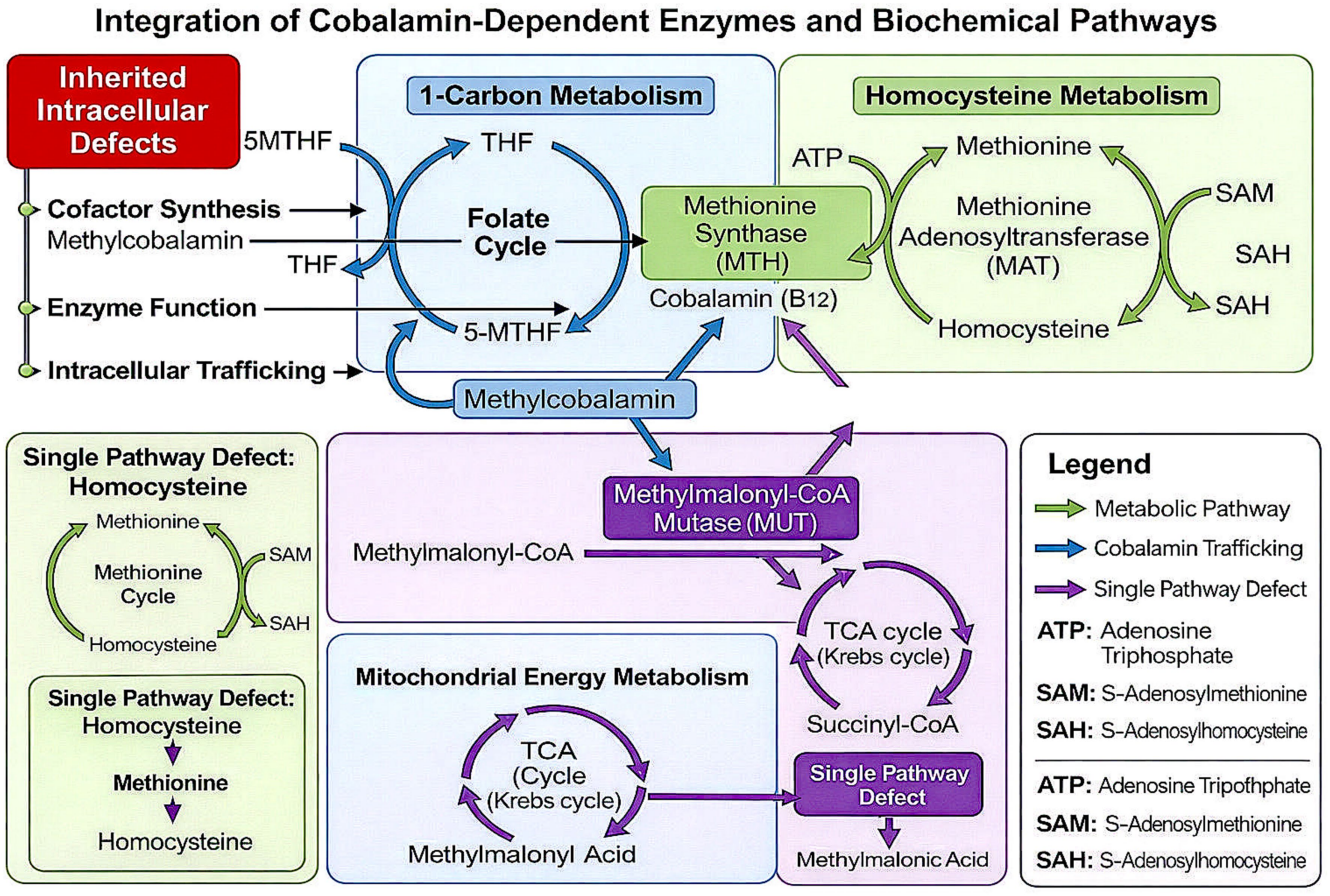
Integration of cobalamin-dependent enzymes and biochemical pathways. Summarizes the role of cobalamin in one-carbon metabolism, homocysteine remethylation, and mitochondrial energy metabolism. Methylcobalamin supports methionine synthase activity linking the folate and methionine cycles, while adenosylcobalamin is required for methylmalonyl-CoA mutase in the conversion of methylmalonyl-CoA to succinyl-CoA in the TCA cycle. Disruption of cobalamin synthesis, function, or intracellular trafficking leads to isolated or combined elevations of homocysteine and methylmalonic acid.

*Mitochondrial energy metabolism:* Methylmalonyl-CoA mutase-mediated conversion of L-methylmalonyl-CoA to succinyl-CoA enables the catabolism of odd-chain fatty acids, cholesterol, and certain amino acids, linking cobalamin metabolism to mitochondrial energy homeostasis. The reaction proceeds via a radical-mediated mechanism involving AdoCbl. These interdependent pathways are depicted in Figure 1.

## 3 Genetic basis of inherited defects in cobalamin metabolism

Inherited defects in cobalamin metabolism are genetically heterogeneous and are classified into ‘complementation groups (cbl)’ - categories of mutations that affect the same gene or functional pathway that cannot ‘complement’ each other in biochemical assays. Mutations within the same complementation group produce the same intracellular biochemical defect, whereas mutations in different groups may cause similar clinical phenotypes but arise from distinct genes or pathways. This classification is critical for accurate diagnosis, genetic counseling, and therapy selection. The disorders can broadly be divided into *intracellular* metabolic defects and *transport or absorption* defects, each leading to distinct biochemical and clinical consequences (Table 1). Inherited intracellular defects impair cofactor synthesis, enzyme function, or intracellular trafficking, affecting either both methylmalonic acid and homocysteine metabolism or a single pathway (12). Intracellular defects often produce a combination of elevated MMA, hyperhomocysteinemia, and low methionine. Defects in uptake, delivery, or binding of cobalamin prevent its availability to cells, producing a functional intracellular deficiency. Transport defects primarily affect intracellular cofactor availability despite normal or high serum B12 levels. The following section provides a brief about these disorders:

### 3.1 Combined methylmalonic aciduria and homocystinuria (MMAHC)

- cblC (MMACHC): The most frequent complementation group, causing defective cytosolic decyanation/dealkylation of dietary cobalamin, which reduces both AdoCbl and MeCbl synthesis (13–15).
- cblD (combined form) (MMADHC): Exhibits mutation-specific effects: N-terminal mutations preferentially impair mitochondrial pathways, whereas C-terminal mutations affect cytosolic methylation (16).
- cblF (LMBRD1) and cblJ (ABCD4): Disrupt lysosomal export of cobalamin, causing intracellular accumulation of unmetabolized B12 and deficiency of both cofactors (17–19).
- cblX (HCFC1) and cblK (ZNF143): Transcriptional defects that indirectly reduce MMACHC expression, leading to combined metabolic deficiency (20–22).

### 3.2 Isolated methylmalonic acidemia (MMA)

- cblA (MMAA) and cblB (MMAB): Mutations impair mitochondrial AdoCbl synthesis or utilization, causing isolated MMA (15).

### 3.3 Isolated homocystinuria (remethylation defects)

- cblE (MTRR) and cblG (MTR): Mutations disrupt MeCbl-dependent methylation of homocysteine to methionine (23). cblE fibroblasts retain near-normal methionine synthase activity under optimal reducing conditions, whereas cblG mutations reduce enzyme activity even under ideal conditions. Clinical features include developmental delay, neurological symptoms, and megaloblastic anemia.

### 3.4 Transport and absorption defects

- Transcobalamin II deficiency (TCN2): Impairs delivery of B12 to tissues, resulting in megaloblastic anemia, pancytopenia, immunodeficiency, failure to thrive, and neurological deficits.
- Transcobalamin receptor deficiency (CD320): Prevents cellular uptake of the holotranscobalamin complex, causing intracellular B12 deficiency, elevated methylmalonic acid, and potential neurological involvement.
- Haptocorrin (TCN1) deficiency: Affects serum B12 binding but generally does not cause clinically significant intracellular deficiency and is considered a biochemical variant.
- Intrinsic factor deficiency (GIF) and Imerslund-Gräsbeck syndrome (CUBN/AMN): Impair intestinal uptake of dietary B12, presenting with megaloblastic anemia, proteinuria, and neurological complications.

## 4 Pathophysiology of neurological injury in cobalamin disorders

### 4.1 Impaired myelination and white matter injury

Cobalamin deficiency disrupts myelin integrity primarily through impaired methionine synthase activity and reduced generation of S-adenosylmethionine (SAM), a critical methyl donor for myelin lipid and protein methylation (24). This leads to demyelination and axonal degeneration, classically manifesting as subacute combined degeneration of the posterior and lateral spinal columns, which may occur despite normal serum vitamin B12 levels when intracellular processing is defective. Impaired methylmalonyl-CoA metabolism further contributes by promoting the incorporation of abnormal fatty acid intermediates into myelin lipids, altering white matter structure.

### 4.2 Neurotoxicity of MMA and homocysteine

The progressive accumulation of methylmalonic acid (MMA) and homocysteine (Hcy) plays a direct role in neuronal injury in cobalamin disorders (25). MMA impairs mitochondrial energy metabolism, reduces ATP production, induces oxidative stress, and promotes neuronal apoptosis, as demonstrated in experimental systems. Elevated homocysteine exacerbates NMDA receptor-mediated excitotoxicity and endothelial dysfunction, further amplifying neuronal damage (26).

### 4.3 Oxidative stress and mitochondrial dysfunction

**Figure 2:**
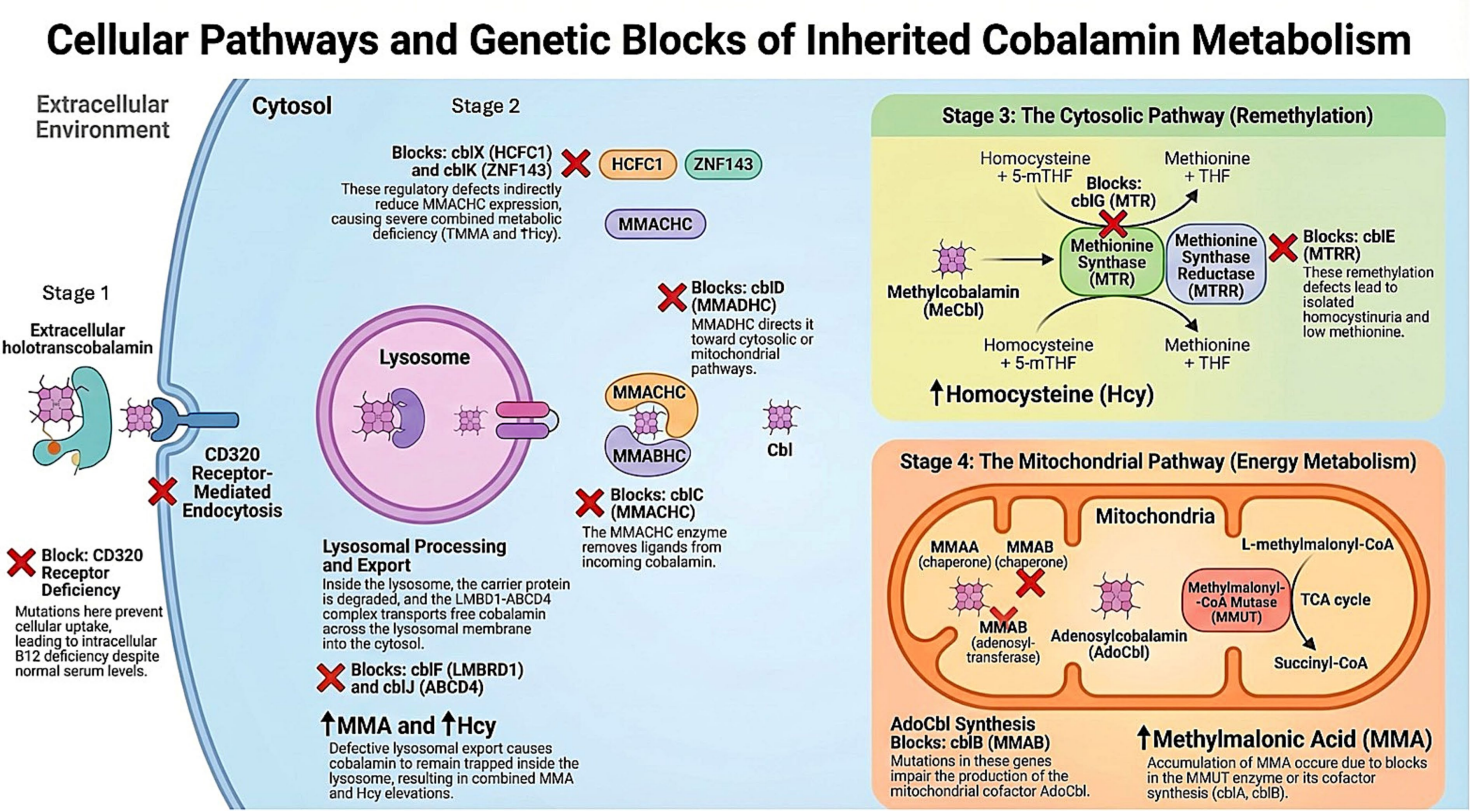
Pathophysiological mechanisms of neurological injury in inherited disorders of intracellular cobalamin metabolism. Illustrates the pathways of neuronal toxicity resulting from genetic defects in cobalamin utilizing enzymes. Stage 1. Cellular entry: Holotranscobalamin enters cells through CD320-mediated endocytosis, then is exported from lysosomes via the ABCD4-LMBD1 complex, with CblJ and CblF linked to defects here. Stage 2. Processing & regulation: MMACHC (CblC) and MMADHC (CblD) direct cobalamin to either cytosolic or mitochondrial pathways, regulated upstream by HCFC1 (CblX) and ZNF143 (CblK). Stage 3. Cytosolic remethylation: Methylcobalamin serves as a cofactor for methionine synthase (MTR, CblG), assisted by MTRR (CblE), converting homocysteine to methionine. Stage 4. Mitochondrial metabolism: Adenosylcobalamin synthesis involves MMAA (CblA) and MMAB (CblB), leading to the MMUT reaction that produces succinyl-CoA for the TCA cycle. Biochemical markers: Remethylation defects elevate homocysteine; mitochondrial pathway defects raise methylmalonic acid.

Mitochondrial dysfunction and oxidative stress represent convergent mechanisms of neurodegeneration in cobalamin disorders (27). Deficiency of adenosylcobalamin-dependent methylmalonyl-CoA mutase activity results in abnormal mitochondrial morphology, ATP depletion, and excessive generation of reactive oxygen species. Experimental studies show that MMA induces lipid peroxidation and compromises antioxidant defenses, leading to progressive neuronal and synaptic injury (Figure 2).

### 4.4 Neurodevelopmental vulnerability in infancy and childhood

The developing brain is particularly vulnerable to cobalamin metabolic disturbances. Early-onset disorders such as cblC commonly present with hypotonia, developmental delay, encephalopathy, and seizures (27). Early and sustained elevations of toxic metabolites are associated with poorer neurodevelopmental outcomes, reflecting heightened susceptibility during critical periods of neuronal maturation and myelination.

## 5 Clinical neurological phenotypes

### 5.1 Neonatal and infantile presentations

Inherited cobalamin metabolism disorders often present early in life, with a spectrum of neurological findings reflecting profound metabolic disruption during brain development. In *cblC* disease, the most common intracellular cobalamin disorder affecting approximately 1 in 100,000–200,000 live births, early-onset cases (≈90%) typically manifest with developmental delay, hypotonia, encephalopathy, and feeding difficulties, often in the first months of life. Systematic studies in early treated children with cblC reveal that nearly half of the cases experienced seizures and more than three-quarters of the children had MRI brain abnormalities by age four, and developmental quotients progressively declined with age in untreated segments, underscoring the impact of metabolic decompensation on neurodevelopment (28, 29). Additional inherited forms like cblD and cblE also feature delayed psychomotor development, hypotonia, seizures, and failure to thrive in infancy, with neurological deficits often persisting despite biochemical correction (Table 2).

**Table 2:** Neurological phenotypes in disorders affecting cobalamin metabolism.

Dominant phenotype	Disorder	Metabolic block	Key neurological features	Key biochemical markers
Neonatal–infantile encephalopathy/developmental delay				
Neonatal-infantile encephalopathy/developmental delay	cblC deficiency	Impaired synthesis of both MeCbl and AdoCbl	Developmental delay, seizures, hypotonia, encephalopathy, microcephaly	↑MMA, ↑Homocysteine, ↓Methionine
	cblD deficiency (combined forms)	Variant-dependent loss of MeCbl and/or AdoCbl	Developmental delay, seizures, hypotonia	↑MMA and/or ↑Homocysteine
	cblF deficiency	Defective lysosomal export of cobalamin	Seizures, developmental delay, encephalopathy	↑MMA, ↑Homocysteine
	cblB deficiency	Defective AdoCbl synthesis	Developmental delay, movement disorder, encephalopathy	↑MMA
	Methylmalonic acidemia (mut^0^/mut^−^)	Absent or reduced methylmalonyl-CoA mutase	Encephalopathy, seizures, metabolic crises	Very ↑MMA, normal homocysteine
Hypotonia, peripheral neuropathy, motor delay				
Hypotonia, peripheral neuropathy, motor delay	cblA deficiency	Impaired AdoCbl synthesis (mitochondrial)	Hypotonia, neuropathy, motor delay	↑MMA
	Transcobalamin II deficiency	Impaired tissue delivery of cobalamin	Developmental delay, neuropathy	Low holo-TC, ↑Homocysteine
Cognitive impairment/neurodevelopmental delay without MMA				
Cognitive impairment/neurodevelopmental delay without MMA	cblE deficiency	Methionine synthase reductase defect	Cognitive impairment, developmental delay	↑Homocysteine, ↓Methionine
	cblG deficiency	Methionine synthase deficiency	Cognitive decline, seizures, hypotonia	↑Homocysteine, ↓Methionine

AdoCbl, adenosylcobalamin; Hcy, homocysteine; HoloTC, holotranscobalamin; MeCbl, methylcobalamin; Met, methionine; MMA, methylmalonic acid.

### 5.2 Childhood and adolescent manifestations

In later childhood, neurological involvement may evolve or persist as cognitive impairment, movement disorders, and behavioural phenotypes. Systematic studies highlight that late-onset *cblC* cases report high frequencies of neuropathy/myelopathy, encephalopathy, including cognitive decline, and psychiatric symptoms as prominent features of disease progression in older children and adolescents (30). In metaseries of inherited defects, most children with confirmed *cblC* exhibited global developmental delay and motor skill deficits, with hypotonia and nystagmus frequently observed, and a subset (25–30%) experienced seizures by early childhood despite treatment (31, 32). Movement abnormalities such as ataxia, dystonia, and extrapyramidal signs have also been reported and may correlate with white matter changes seen on neuroimaging (33) (Table 2).

## 6 Neuroimaging and neurophysiological findings

### 6.1 MRI patterns in cobalamin disorders

**Figure 3:**
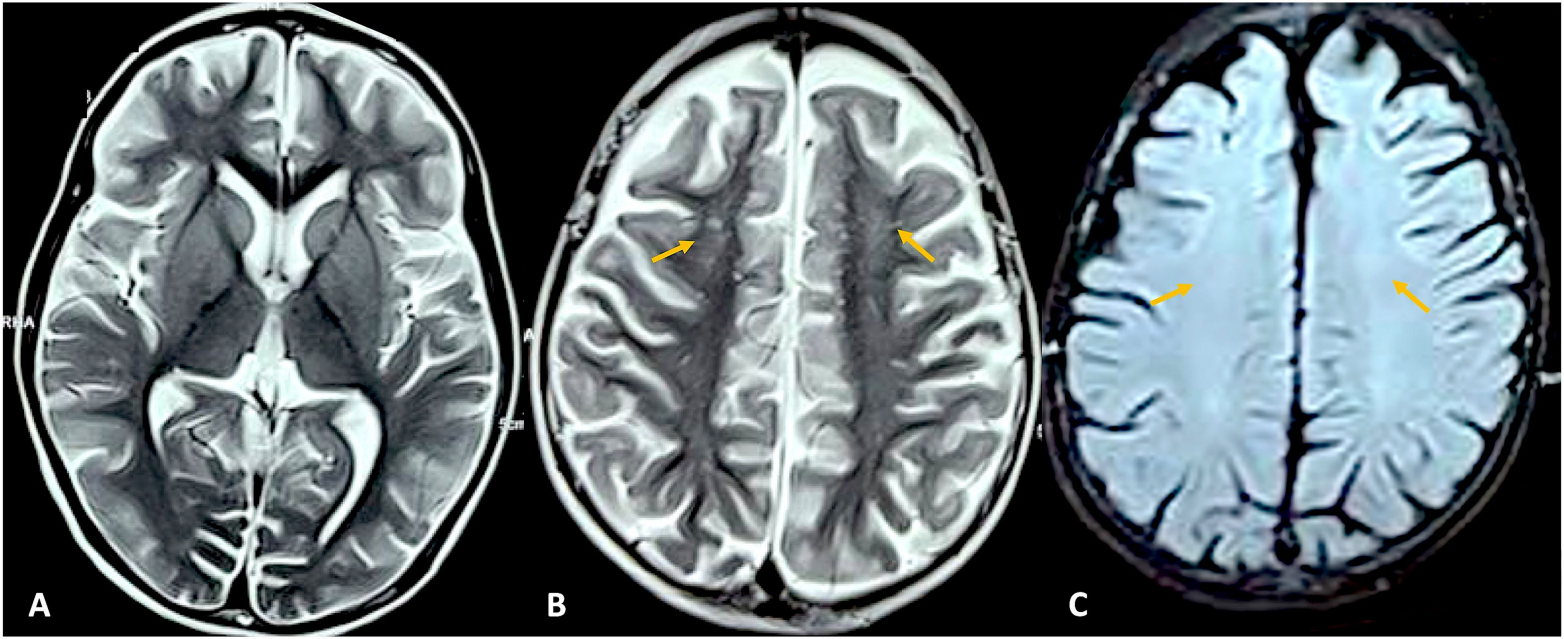
Neuroimaging in MMACHC. MRI brain axial sections T2-weighted sequences **(A)** showing diffuse brain atrophy, sparing of basal ganglia, and T2-weighted **(B)** and FLAIR **(C)** sequences showing hyperintensities in the fronto-parietal white matter bilaterally in a child with MMACHC.

Cobalamin is essential for myelin development and cellular methylation. Disorders of cobalamin and folate metabolism are established causes of leukoencephalopathy in pediatric populations. In infants and children with acquired cobalamin deficiency, brain MRI typically shows delayed or arrested myelination, diffuse cerebral atrophy with ventriculomegaly, thinning of the corpus callosum, and symmetric periventricular or subcortical white-matter T2/FLAIR hyperintensities (34). These findings reflect impaired myelin formation and are often partially reversible with treatment (35). Inherited intracellular cobalamin disorders, particularly remethylation defects such as cobalamin C (cblC) disease, show a more severe pediatric neuroimaging phenotype. MRI commonly demonstrates progressive periventricular and deep white-matter abnormalities, corpus callosum thinning, and cerebral and cerebellar atrophy, consistent with leukodystrophy (Figure 3) (36). A key distinguishing feature in children is bilateral involvement of the deep gray nuclei, most often the globus pallidus, putamen, caudate nuclei, and thalami, reflecting selective vulnerability in remethylation disorders. Spinal cord involvement resembling subacute combined degeneration, with symmetric posterior column T2 hyperintensity, has also been reported and may improve with metabolic therapy.

### 6.2 MR spectroscopy and advanced imaging

Advanced MRI techniques provide additional insight into metabolic dysfunction in pediatric cobalamin-related disorders. Proton MR spectroscopy in children with methylmalonic acidemia and remethylation defects has shown elevated lactate, reduced N-acetylaspartate, and altered myoinositol, consistent with impaired oxidative metabolism and neuronal injury. These abnormalities may precede structural changes on conventional MRI. Recent pediatric cohort studies indicate that conventional MRI markers, including ventricular dilation, corpus callosum thinning, and cerebral atrophy, are associated with poorer neurodevelopmental outcomes in infants with cblC defects (36, 37). Combined use of structural, diffusion, and spectroscopic imaging may improve early detection and prognostic stratification.

### 6.3 Electroencephalographic (EEG) and neurophysiological studies

EEG abnormalities are common in children with cobalamin metabolism disorders, particularly early-onset cblC disease and MMA. Typical findings include diffuse background slowing and focal or multifocal epileptiform discharges, often associated with seizures and encephalopathy, and sometimes present despite normal neuroimaging (38–40). Nerve conduction studies in chronic or severe deficiency may demonstrate axonal sensorimotor neuropathy, reflecting combined central and peripheral involvement (41). Early EEG abnormalities should prompt metabolic evaluation, as treatment may improve neurophysiological outcomes.

## 7 Diagnostic approach

### 7.1 First-tier biochemical testing

Initial investigations include

- Complete blood count with peripheral smear: Evaluates red blood cell indices and classical hematological manifestations of cobalamin deficiency, including megaloblastic and pernicious anemia (42, 43). Also, leukopenia and thrombocytopenia can result from bone marrow suppression (44).
- Basic metabolic profile: To look for electrolyte abnormalities, liver, and renal function tests.
- Venous blood gas: To identify metabolic acidosis, which can be seen with methylmalonic acidemia.
- Serum total vitamin B12 (holo-transcobalamin) levels: The most widely used biomarker of cobalamin status (45). Plasma/serum methylmalonic acid levels are the functional and non-sensitive biomarker of cobalamin status (45, 46). It can also be elevated with impaired renal function (45).
- Serum total homocysteine levels: Also, a functional biomarker of cobalamin status. It is also noted to be elevated in vitamin B6 deficiency, folate deficiency, impaired renal function, and hypothyroidism (47).

Elevated serum methylmalonic acid and/or total homocysteine levels may confirm the diagnosis of cobalamin deficiency; however, in symptomatic patients, normal levels do not exclude this diagnosis. Serum methionine, folate, and pyridoxine levels may also be done as indicated. Urine analysis to look for proteinuria.

### 7.2 Second-tier biochemical testing

Acylcarnitine profiling by tandem mass spectrometry (MS/MS) establishes the acylcarnitine profile. Urine acylcarnitine levels can be determined by gas chromatography–mass spectrometry (GC–MS). Elevated propionylcarnitine (C3) and an elevated C3/C2 ratio are characteristic findings in methylmalonic acidemia and related cobalamin disorders.

### 7.3 Genetic testing

Genetic testing is crucial to diagnose these disorders, especially late-onset disorders, and also in identifying asymptomatic carriers through testing of the relatives. The targeted sequencing with a single gene or multigene panel, or whole exome sequencing (WES) is done to identify the biallelic pathogenic variants in the genes summarized in Table 2.

### 7.4 Newborn screening

The newborn screening for cobalamin disorders is done by measuring the acylcarnitines C3 (propionylcarnitine) and methionine by dried blood spot testing through tandem mass spectrometry (MS/MS). Propionylcarnitine (C3) levels and the C3/C2 (acetylcarnitine) ratio are commonly elevated markers in NBS (48, 49). These markers are elevated in the disorders of methymalonic acidemia, propionic acidemia, and other disorders of cobalamin metabolism (50). Further secondary testing of analytes are needed for differentiating these disorders. Late-onset cobalamin disorders may not be picked up through NBS, as the first symptoms and metabolic alterations usually occur after 12 months of life (30).

### 7.5 Other investigations

**Figure 4:**
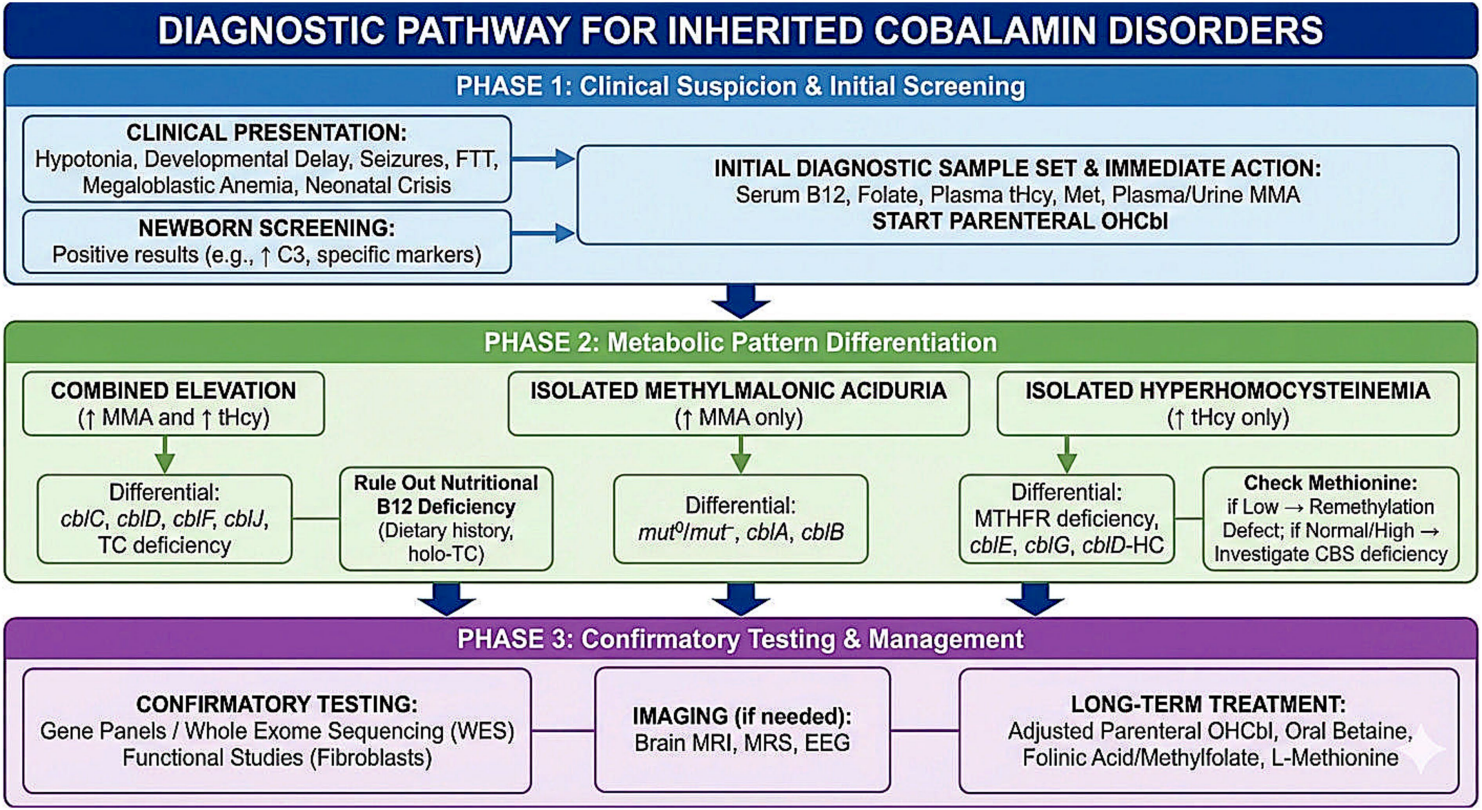
Diagnostic pathway for inherited cobalamin disorders. Outlines a three-phase approach to diagnosing inherited disorders of cobalamin metabolism, moving from initial suspicion to confirmatory testing and management.

Additional investigations may include gastric pH monitoring, anti-intrinsic factor antibodies, and the Schilling test for evaluation of intrinsic factor deficiency. Bone marrow examination may show megaloblastic erythropoiesis, hypersegmented neutrophils, dysplastic changes, and pancytopenia. Severe cobalamin deficiency can mimic and also co-exist in myelodysplastic syndromes (51). Prenatal diagnosis by targeted molecular testing is feasible when pathogenic variants are known in parents. The diagnostic pathway for inherited cobalamin disorders is summarised in Figure 4.

## 8 Differential diagnosis

In neonates, children, and adolescents, biochemical or neurological findings suggestive of cobalamin deficiency frequently reflect secondary disruption of cobalamin-dependent pathways rather than true cobalamin deficiency (52). These mimics arise from mitochondrial dysfunction, impaired one-carbon metabolism, altered metabolite clearance, or functional enzyme inhibition, leading to laboratory abnormalities that overlap with cobalamin deficiency.

Clinically, mild or transient elevations of MMA are common in pediatric patients with inborn errors of energy metabolism, mitochondrial disease, intercurrent illness, or reduced renal clearance and should not be interpreted in isolation. Isolated homocysteine elevation with normal MMA generally indicates impaired remethylation rather than cobalamin deficiency, while marked homocysteine elevation with normal MMA suggests a non-B_12_-mediated block such as transsulfuration defects or functional cobalamin inactivation (53, 54). Diagnosis depends on biochemical pattern recognition, age at presentation, nutritional and exposure history, and evidence of multisystem involvement. Serum B12 concentrations may be normal or misleading and should not be used alone to establish the diagnosis. Early distinction between true B12 deficiency and secondary mimics is essential (Table 3) (53, 54).

**Table 3:** Pediatric and adolescent disorders mimicking cobalamin deficiency.

Disease	Mechanism	Neurological effects	Key biochemical effects
Inherited disorders			
Organic acidemias (e.g., propionic acidemia)	Propionyl-CoA accumulation interferes with methylmalonyl-CoA metabolism	Encephalopathy, hypotonia, developmental delay	Mild–moderate ↑MMA, normal B12
Fatty acid oxidation disorders (VLCAD, MADD)	Secondary mitochondrial dysfunction impairs AdoCbl-dependent reactions	Lethargy, encephalopathy, metabolic crises	Mild ↑MMA
TCA cycle disorders (SUCLA2, SUCLG1 deficiency)	Succinyl-CoA depletion disrupts methylmalonyl-CoA flux	Hypotonia, developmental delay	Mild ↑MMA
mtDNA maintenance disorders (POLG, MPV17, RRM2B)	Global mitochondrial dysfunction reduces cobalamin utilization	Neuropathy, myopathy, seizures	Mild ↑MMA
Folate pathway disorders (e.g., MTHFR deficiency)	Impaired remethylation of homocysteine	Developmental delay, cognitive slowing	↑Homocysteine, normal MMA
Transsulfuration defects (CBS deficiency)	Blocked conversion of homocysteine to cystathionine	Developmental delay, lens dislocation, thrombosis	Markedly ↑Homocysteine, normal MMA

MMA, methylmalonic acid; TCA, tricarboxylic acid.

## 9 Therapeutic strategies

**Figure 5:**
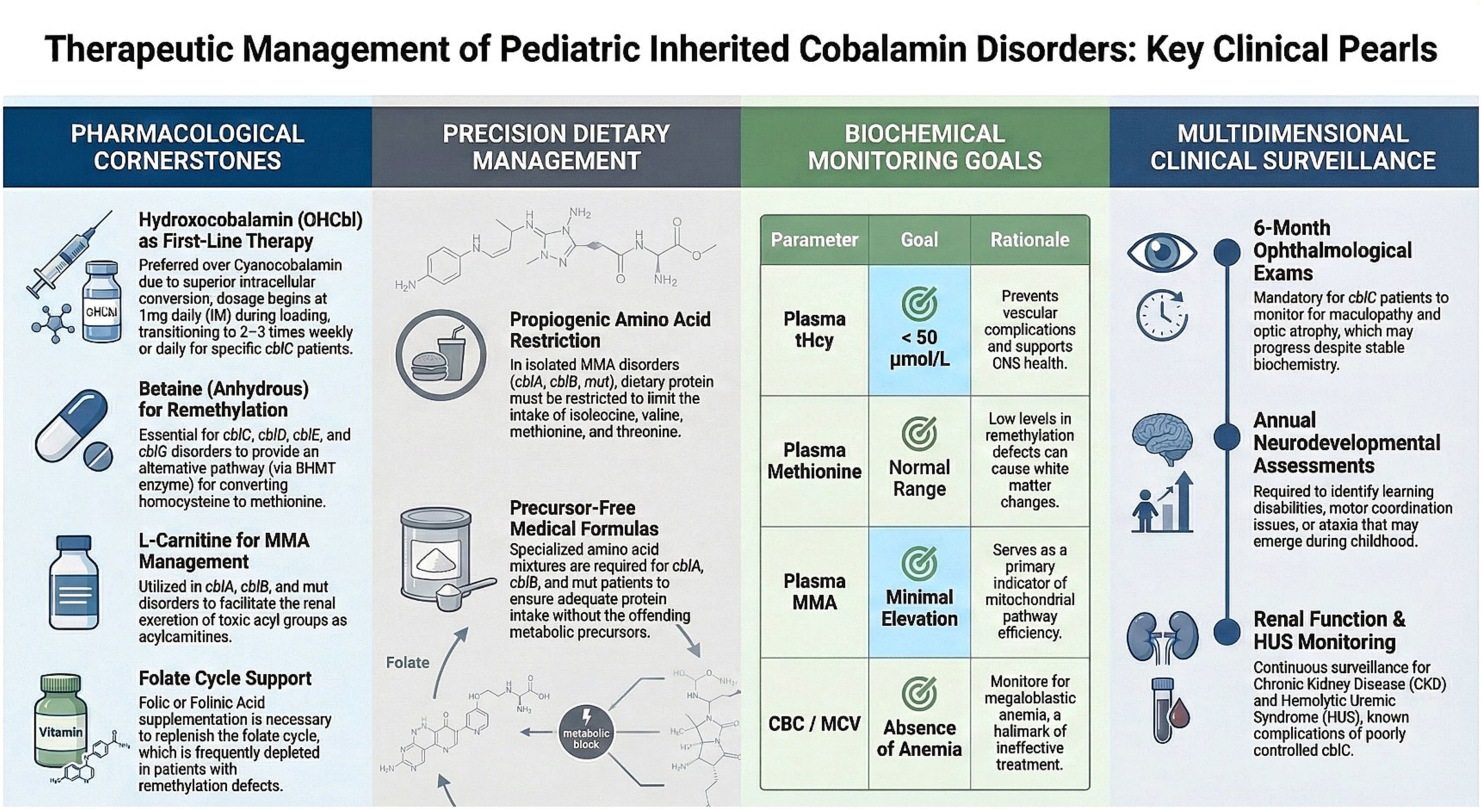
Key clinical pearls. Provides a central illustration of key clinical pearls in therapeutic management, summarizing dosing regimens, administration routes (parenteral versus oral), and monitoring parameters.

The management of inherited cobalamin disorders requires targeted supplementation with the appropriate cobalamin form, combined with adjunctive metabolic therapies tailored to the specific complementation group. Figure 5 provides a central illustration of key clinical pearls in therapeutic management, summarizing dosing regimens, administration routes (parenteral versus oral), and monitoring parameters. The therapeutic management is summarised in Table 4.

**Table 4:** Therapeutic management summary.

Disorder category	Primary therapy (dose/route)	Adjunctive therapies
Combined defects (cblC, cblF, cblJ)	Parenteral hydroxocobalamin (OHCbl) 1 mg/day IM/SC	Betaine (250 mg/kg/day), carnitine, folinic acid (15 mg 4x daily). Oral OHCbl is ineffective for cblC.
Isolated MMA (cblA, cblB)	OHCbl 1 mg IM (standardized 3-day test)	Protein-restricted diet. Carnitine (50–100 mg/kg/day).
Remethylation defects (cblE, cblG)	OHCbl or methylcobalamin 1 mg daily, then weekly	Betaine (250 mg/kg/day), folinic acid, L-methionine (40 mg/kg/day for cblG).
Transport defects (TCN2)	OHCbl 0.5-1 mg daily (Initial), then twice weekly	Folinic acid (15 mg PO 4x daily). IV B12 is not recommended due to rapid urinary loss
Absorption defects (GIF, IGS)	OHCbl 1 mg/day IM until values normalize, then 0.25 mg every 3 months	Monitor for proteinuria (IGS) and secondary nutritional deficiencies

MMA, methylmalonic acid.

### 9.1 Disorders of absorption and transport of cobalamin

- Hereditary intrinsic factor deficiency, Imerslund-Gräsbeck syndrome: Hydroxocobalamin is used at 1 mg/day intramuscular until biochemical and hematological values normalize, followed by doses of 0.25 mg every 3 months to maintain levels.
- Transcobalamin deficiency: Oral or parenteral hydroxocobalamin or cyanocobalamin at 0.5-1 mg, initially daily, and then twice weekly to maintain levels. Intravenous cobalamin is not recommended due to rapid loss in urine.

### 9.2 Disorders of intracellular utilization of cobalamin

- Adenosylcobalamin deficiency (CblA and CblB types): A standardized protocol with administration of 1 mg hydroxocobalamin for 3 days, with a reduction in plasma or urine MMA of ≥ 50% over 10 days, is considered a positive response. Further therapy is with 10 mg P.O or 1 mg I.M once or twice weekly, although poor response is noted with CblB patients with this regimen.
- Methylcobalamin deficiency (CblE and CblG types): Treated with hydroxocobalamin or methylcobalamin at 1 mg, initially daily, followed by once or twice weekly.
- Combined deficiencies of adenosylcobalamin and methylcobalamin: For the CblC group, parenteral hydroxocobalamin is used at 1 mg/day, and oral hydroxocobalamin, folinic acid, and carnitine are ineffective. CblF and CblJ types are treated with parenteral hydroxocobalamin at 1 mg/day, initially daily, and then biweekly. CblD types are treated based on the subtypes and are similar to CblC, CblA/B, and CblE/G types.

### 9.3 Adjunctive metabolic therapies (betaine, folate, carnitine, etc.)

- Carnitine: 50-100 mg/kg/day for CblA and CblB types.
- Betaine: 250 mg/kg/day for CblE, CblG, and CblC types.
- Folic acid and folinic acid: Doses up to 15 mg P.O four times daily have been used in transcobalamin deficiency. Folates must not be used as the only therapy in transcobalamin deficiency. Folic acid and folinic acid are used in CblE and CblG types.
- L-methionine: 40 mg/kg/day has been used in CblG types.
- Protein-restricted diet: Most of the CblA and CblB patients respond to protein restricted diet.
- Organ transplantation: Renal transplantation may be done in end-stage renal disease. Liver transplantation may be done to prevent metabolic decompensation.

### 9.4 Dietary management

- Protein restriction: In isolated MMA disorders (cblA, cblB, mut), a medical diet low in propiogenic amino acids (isoleucine, valine, methionine, threonine) is necessary to prevent the accumulation of MMA.
- Precursor-free Formulas: Specialized amino acid mixtures provide essential protein without the offending precursors.

### 9.5 Timing of treatment

Early diagnosis is essential to prevent irreversible damage to the developing brain caused by impaired myelination, white matter injury, neurotoxicity, and oxidative stress with mitochondrial dysfunction. The goals of management are to halt the progression of cognitive impairment, neurodevelopmental delay, recurrent infections, feeding difficulties, and seizures, and possibly prevent life-threatening metabolic crises and reverse deficits. Newborn screening has an important role in the early detection and treatment initiation, and may prevent the development of brain lesions and promote a favorable neurodevelopment in cobalamin defects (28, 55, 56).

Missing the critical window for therapeutic interventions will result in irreversible damage and permanent disabilities. The irreversibility of symptoms due to delayed diagnosis and treatment initiation can result in end-stage renal disease, liver failure, cardiomyopathy, failure to thrive, respiratory failure, pulmonary hypertension, renal thrombotic microangiopathy, myelopathy from spinal cord degeneration, cerebral atrophy, stroke, seizures, and coma (30, 57, 58).

## 10 Prognosis and anticipated outcomes

The prognosis and long-term outcomes vary considerably across subtypes of inherited cobalamin disorders. The cblC group has been most extensively studied as the most common subtype. Despite hydroxocobalamin treatment, long-term outcomes in children with cblC disease are frequently unsatisfactory (30, 59, 60). The biochemical abnormalities, non-neurological symptoms, and mortality may improve with medical management, while the long-term ophthalmological and neurological outcomes often remain unchanged (30, 59).

- Biochemical response: Most patients show a rapid decline in plasma Homocysteine (tHcy) and Methylmalonic acid (MMA) within days of starting high-dose OHCbl. Complete normalization is rare in cblC, but “safe” levels are usually achievable.
- Neurological stability: Early treatment prevents the most severe forms of encephalopathy and developmental delay. However, neurological “flares” can still occur during illness.
- Ophthalmological challenges: In cblC deficiency, maculopathy and optic atrophy are notoriously resistant to systemic therapy. Even with excellent biochemical control, vision may continue to deteriorate.
- Survival: Modern management has transformed these from fatal neonatal conditions to chronic, manageable diseases.

### 10.1 Factors influencing recovery

Factors influencing recovery in both acquired and hereditary cobalamin disorders may include the underlying etiology, age at diagnosis, clinical severity at presentation, severity and duration of metabolic decompensation, timing of treatment initiation, adherence to therapy, and multi-disciplinary follow-up (33, 61–63).

### 10.2 Neurodevelopmental follow-up

Among predictors of outcome, brain lesions and epilepsy were reported to be associated with poor neurodevelopmental outcomes (28, 35, 64). Ricci et al. studied neurodevelopmental characterization in children with CblC defect and noted a specific fall in language development after 24 months in CblC defect (28). While Weisfeld-Adams et al. noted motor-predominant developmental delay in their cohort of young children with CblC type (32). Multimodal neurodevelopmental surveillance incorporating cognitive, motor, language, and ophthalmological assessments, is recommended for all affected children from the time of diagnosis.

### 10.3 Monitoring and long-term management

Monitoring must be lifelong and multidimensional, combining biochemical tracking with clinical surveillance (Table 5).

**Table 5:** Biochemical monitoring (frequency: every 3–6 months).

Parameter	Goal	Rationale
Plasma tHcy	<50 μmol/L	Prevents vascular complications and supports CNS health.
Plasma MMA	Minimal elevation	Reflects mitochondrial pathway efficiency.
Plasma methionine	Normal range	Low methionine (in remethylation defects) causes white matter changes.
CBC/MCV	Absence of anemia	Megaloblastic anemia is a hallmark of ineffective treatment.

CBC, complete blood count; Hcy, homocysteine; MCV, mean corpuscular volume, MMA, methylmalonic acid.

Clinical surveillance

- Ophthalmology: Formal eye exams every 6 months for cblC patients to monitor for pigmentary retinopathy.
- Neurodevelopmental testing: Annual assessments to identify learning disabilities or motor coordination issues (ataxia) early.
- Renal function: Monitoring for Chronic Kidney Disease (CKD) or Hemolytic Uremic Syndrome (HUS), which are common complications of poorly controlled cblC.

## 11 Emerging concepts and future directions

### 11.1 Novel genetic and epigenetic mechanisms

Recent studies demonstrate that cobalamin metabolism disorders can arise from transcriptional and regulatory dysfunction, not only enzymatic defects. The cblX disorder is caused by pathogenic variants in *HCFC1*, an X-linked transcriptional coregulator required for *MMACHC* expression, resulting in combined methylmalonic acidemia and hyperhomocysteinemia with severe neurodevelopmental impairment and early-onset epilepsy (20). Variants in *ZNF143*, a transcription factor that interacts with HCFC1, similarly reduce *MMACHC* transcription and define a cblX-like regulatory disorder, confirming impaired gene regulation as a disease mechanism (22). Recent genetic models using advanced perturbation tools further demonstrate that disruption of *MMACHC* regulatory networks alters mammalian development beyond canonical cobalamin metabolism, implicating transcriptional and epigenetic control as central drivers of neurological pathology (65).

### 11.2 Biomarkers of early neurological injury

Early neurological injury in cobalamin disorders is best detected using integrated biochemical and imaging biomarkers (30, 66). Plasma MMA and total homocysteine remain the most sensitive biochemical indicators, often rising before clinical symptoms (66, 67), while holotranscobalamin (HoloTC) better reflects biologically active cobalamin and improves diagnostic sensitivity compared with total B₁₂ alone (68, 69). Emerging evidence also highlights neuroimaging signatures-including subclinical white-matter changes on MRI-as surrogate markers of early neural injury (70). Multimodal integration of metabolic and imaging biomarkers can enable earlier intervention to prevent irreversible neurological damage.

### 11.3 Gene therapy and precision medicine

As the genetic architecture of cobalamin metabolism disorders expands, precision medicine strategies are increasingly relevant (71). Although gene therapy remains investigational, preclinical studies using adeno-associated viral vectors and CRISPR-dependent base editing have shown proof of concept for restoring *MMACHC* expression and correcting pathogenic variants in cellular and animal models, including regulatory defects such as *HCFC1*-linked cblX. Clinically, genotype-informed management now guides hydroxocobalamin dosing and adjunctive therapies, with biomarker-guided monitoring (MMA, homocysteine, neuroimaging changes) optimizing neurologic outcomes in high-risk genotypes. Advances in molecular diagnostics and targeted small molecules promise genotype-specific modulation for patients with suboptimal response to conventional therapy (72).

## 12 Clinical challenges and unmet needs

In low- and middle-income countries (LMICs), inherited cobalamin metabolism disorders remain underdiagnosed due to limited access to newborn screening, metabolic assays, and genetic testing (73). Children often present late with non-specific neurological features, including seizures or developmental regression, which are frequently attributed to infectious or nutritional causes (29). Restricted availability of MMA and homocysteine testing, parenteral hydroxocobalamin, and long-term multidisciplinary care contributes to delayed treatment and irreversible neurological injury. Transition of care into adolescence and adulthood, management of attenuated phenotypes, and assessment of long-term cognitive and psychiatric outcomes remain unmet needs (74).

In high-income countries, challenges relate primarily to diagnostic complexity and evidence gaps. Milder or late-onset phenotypes may be missed by newborn screening thresholds optimized for severe disease (75), while phenotypic heterogeneity can delay metabolic evaluation. After diagnosis, limited data guide optimal treatment intensity, adjunctive therapies, and long-term neurodevelopmental follow-up.

## 13 Conclusion

Cobalamin metabolism is a complex but essential system that integrates one-carbon transfer reactions with mitochondrial energy homeostasis to maintain neurological integrity. Disruptions in these pathways – whether due to acquired deficiencies or rare inborn errors – result in a diverse spectrum of clinical phenotypes in children, ranging from neonatal encephalopathy and developmental delay to progressive cognitive and motor decline. While the genetic etiology of the most prevalent subtypes has been largely elucidated, significant gaps persist regarding the interactome governing intracellular cobalamin trafficking, and the profound genotype–phenotype discordance observed across patient cohorts suggests modulation by yet-unidentified epigenetic regulators or modifier genes. The primary drivers of neurological injury are demyelination, global hypomethylation, and the neurotoxic accumulation of homocysteine and methylmalonic acid. Because conventional serum B₁₂ levels can be misleading, a diagnostic strategy utilizing MMA, homocysteine, and holotranscobalamin is necessary for early detection. Prompt and aggressive intervention is the most critical factor in halting disease progression and preventing irreversible damage to the developing brain. Future research must prioritize integration of multi-omic datasets – specifically transcriptomics and proteomics - to identify secondary modifiers and clarify metabolic flux under varying physiological stresses. Development of patient-derived induced pluripotent stem cell (iPSC) and organoid models is essential for studying tissue-specific manifestations, particularly within the neuro-renal axis. Therapeutically, the field is moving toward precision interventions including mRNA-based therapies, small-molecule chaperones, and CRISPR-Cas9-mediated gene correction. Establishing longitudinal registries to validate high-sensitivity biomarkers is imperative to transition the standard of care from reactive management to proactive, precision-based prevention of irreversible neurological sequelae.
