# Supplementary material for: Inherited disorders of cobalamin metabolism in childhood: biochemical and clinical perspectives
Source: Front Nutr. 2026 May 4;13:1808765. doi: 10.3389/fnut.2026.1808765 (PMC13180602; doi:10.3389/fnut.2026.1808765)
Supplement: Supplementary file 1 [file Table_1.DOCX]

## Supplement

## Supplement 1: Timeline of key foundational discoveries in inherited disorders of cobalamin metabolism (1970-2010)

| **Complementation Group*** | **Major Phenotype** | **Context/Mechanism Delineated** | **Year** |
| --- | --- | --- | --- |
| CblC (Phenotype) | Combined MMA & Hcy | Initial biochemical recognition of combined methylmalonic acidemia and homocystinuria due to impaired cobalamin coenzyme synthesis^6^ | ~1970 |
| CblC (Designation) | Combined MMA & Hcy | Formal definition of the cblC genetic complementation group via fibroblast heterokaryon studies^7^ | 1975 |
| CblA/ CblB | Isolated MMA (vitamin B12–responsive) | Delineation of cblA and cblB as distinct complementation groups affecting adenosylcobalamin synthesis^8^ | 1978 |
| CblE / CblG | Isolated Hcy | Identification of methionine synthase defects through enzyme activity assays and complementation analysis | Early 1980s |
| CblF | Combined MMA & Hcy | Definition of a complementation group characterized by defective lysosomal release of cobalamin^9^ | 1986 |
| CblD | Variable MMA/Hcy | Molecular identification of MMADHC defining the cblD complementation group^10^ | 2008 |
| CblJ | Combined MMA & Hcy | Identification of ABCD4 defect causing secondary lysosomal cobalamin transport failure^11^ | 2010 |

*Complementation group nomenclature reflects functional classification based on cellular complementation studies and does not necessarily correspond to the chronological order of gene discovery.
